# 2507 A novel multi-photon microscopy method for neuronavigation in deep brain stimulation surgery

**DOI:** 10.1017/cts.2018.40

**Published:** 2018-11-21

**Authors:** Nicholas M. George, Arianna G. Polese, Greg Futia, Baris Ozbay, Wendy Macklin, Emily Gibson, Aviva Abosch, Diego Restrepo, Brian E. Moore

**Affiliations:** 1 Department of Pathology, University of Colorado, Anschutz Medical Campus

## Abstract

OBJECTIVES/SPECIFIC AIMS: The goal for this project is to determine the feasibility of using a novel multi-photon fiber-coupled microscope to aid surgeons in localizing STN during surgeries. In order to accomplish this goal, we needed to identify the source of a strong autofluorescent signal in the STN and determine whether we could use image classification methods to automatically distinguish STN from surrounding brain regions. METHODS/STUDY POPULATION: We acquired 3 cadaveric brains from the University of Colorado Anschutz Medical Campus, Department of Pathology. Two of these brains were non-PD controls whereas 1 was diagnosed with PD. We dissected a 10 square centimeter region of midbrain surrounding STN, then prepared this tissue for slicing on a vibratome or cryostat. Samples were immuno-labeled for various cellular markers for identification, or left unlabeled in order to observe the autofluorescence for image classification. RESULTS/ANTICIPATED RESULTS: The border of STN is clearly visible based on the density of a strong autofluorescent signal. The autofluorescent signal is visible using 2-photon (850–1040 nm excitation) and conventional confocal microscopy (488–647 nm excitation). We were also able to visualize blood vessels with second harmonic generation. The autofluorescent signal is quenched by high concentrations of Sudan-black B (0.5%–5%), and is primarily localized in microtubule-associated protein-2 (MAP2)+ cells, indicating that it is likely lipofuscin accumulation in neurons. Smaller lipofuscin particles also accumulate in microglia, identified based on ionized calcium binding adopter 1 (Iba1)+ labeling. We anticipate that colocalization analysis will confirm these qualitative observations. Using 2-photon images of the endogenous autofluorescent signal in these samples, we trained a logistic regression-based image classifier using features derived from gray-level co-occurrence matrices. Preliminary testing indicates that our classifier performed well, with a mean accuracy of 0.89 (standard deviation of 0.11) and a Cohen’s Kappa value of 0.76 (standard deviation of 0.24). We are currently using coherent anti-Stokes Raman scattering and third harmonic imaging to identify different features of myelin that can be used to distinguish between these regions and expect similar results. DISCUSSION/SIGNIFICANCE OF IMPACT: Traditional methods for localizing STN during DBS surgery include the use of stereotactic coordinates and multi-electrode recording (MER) during implantation. MERs are incredibly useful in DBS surgeries, but require penetration of brain structures in order to infer location. Using multi-photon microscopy techniques to aid identification of STN during DBS surgeries offers a number of advantages over traditional methods. For example, blood vessels can be clearly identified with second harmonic generation, something that is not possible with MER. Multi-photon microscopy also allows visualization deep into tissue without actually penetrating it. This ability to look within a depth of field is useful for detection of STN borders based on autofluorescent cell density. When combined with traditional stereotactic information, our preliminary image classification methods are a fast, reliable way to provide surgeons with extra information concerning their location in the midbrain. We anticipate that future advancements and refinements to our image classifier will only increase accuracy and the potential applications and value. In summary, these preliminary data support the feasibility of multi-photon microscopy to aid in the identification of target brain regions during DBS surgeries. The techniques described here complement and enhance current stereotactic and electrophysiological methods for DBS surgeries.

